# Intra‐CSF topotecan in treatment of breast cancer patients with leptomeningeal metastases

**DOI:** 10.1002/cam4.3422

**Published:** 2020-09-03

**Authors:** Kurt A. Jaeckle, Jesse G. Dixon, Stephen Keith Anderson, Alvaro Moreno‐Aspitia, Gerardo Colon‐Otero, Kathy Hebenstreit, Tejal A. Patel, Samarth L. Reddy, Edith A. Perez

**Affiliations:** ^1^ Mayo Clinic Florida Jacksonville FL USA; ^2^ Mayo Clinic Rochester Rochester MN USA; ^3^ Houston Methodist Hospital Cancer Center Houston TX USA; ^4^ Hematology Oncology Associates of Boca Raton Boca Raton FL USA

**Keywords:** breast cancer, leptomeningeal metastasis, neoplastic meningitis, topotecan

## Abstract

**Background:**

There are few treatment options for patients with leptomeningeal metastases (LM).

**Methods:**

We report a case series of patients with breast cancer and LM treated with intra‐CSF topotecan (TOPO). Outcome was assessed by clinical exam and MRI at baseline, at end of induction (4‐5 weeks), then every 3 months; CSF cytology was determined at baseline and with each treatment.

**Results:**

Thirty‐one women [median age, 58 (37‐81); median KPS 60 (40‐100)] received treatment. At baseline, 68% had positive CSF cytology, and 90%, leptomeningeal enhancement on MRI. 84% of patients also received focal RT (not during TOPO) and 77% received concomitant systemic hormonal or chemotherapy. Median number of TOPO treatments was 14.5 (range, 3‐71); median duration of treatment, 11 weeks (1‐176); and median OS, 6.9 months (range, 0.9‐48.8). Patients remaining progression‐free during 4‐6 weeks of induction (81%) had a median OS of 11.5 months (range, 1.8‐48.8). Overall neurologic PFS at 6, 12, and 24 months was 39%, 26%, and 6%, respectively. Clearing of CSF malignant cells for >3 consecutive samples occurred in 10/21 (48%) patients with positive CSF cytology at baseline, remaining clear for a median duration of 15.9 months (range, 1.4‐34.5). Grade 3 adverse events included headache or vomiting (3pts), T2 hyperintensity surrounding the ventricular catheter (2 pts), and meningitis (2 pts).

**Conclusions:**

Intra‐CSF TOPO, with focal RT as needed for symptomatic areas of enhancement produced durable clearing of CSF malignant cells in 48% of patients positive at baseline, with promising median PFS and OS.

## Graphical abstract

In this case series, intra‐CSF topotecan treatment of women with leptomeningeal metastases and breast cancer was associated with durable CSF clearing of malignant cells in 48% of patients, median OS of 6.9 months, and 11.5 months in those completing 4‐6 weeks induction. Thirty‐two percentage of patients remained free of neurologic progression at 12 months. A method for treatment of patients with VP shunts and programmable valves is also described.

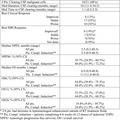

## 1 INTRODUCTION

Treatment options for patients with leptomeningeal metastases (LM) are limited, and survival after diagnosis is typically 2‐3 months.^1^ Available drugs for intra‐CSF clinical use include methotrexate, cytarabine, and thio‐TEPA, but these agents have shown limited efficacy. Clearly, newer and effective therapies are needed.

Topotecan (TOPO) is a semi‐synthetic water‐soluble topoisomerase 1 inhibitor which is approved by the US Food and Drug Administration for treatment of refractory ovarian and metastatic small cell lung carcinoma, and in combination with cisplatin for refractory Stage IVB Topotecan also has activity in breast cancer, with objective responses observed following single‐agent treatment in 10% (95% CI: 3,24%) of patients, but significant associated hematologic toxicity (62% CTC Grade ≥3) has limited this application.^2^, ^3^ Given that a 450‐fold increase in CSF concentration can be achieved with intra‐CSF administration at 1% of the systemically administered dose, and without added hematologic toxicity,^4^, ^5^ the intra‐CSF route of administration has been explored for treatment of LM, showing limited activity in solid‐tumor malignancies,^4^ but more promising activity in childhood leukemia^6^, ^7^ and CNS gliomas.^8^ We now report a case series review of breast cancer patients with LM treated at our institution, with the hypothesis that promising activity might be observed in this specific cohort.

## 2 METHODS

We performed a retrospective review of clinical records and imaging studies from breast cancer patients with LM treated with intra‐CSF TOPO prospectively (2006‐2016; data lock 7/15/20). LM was defined as: clinical symptoms and/or exam findings consistent with LM; and either positive CSF cytology for malignant cells at baseline, or evidence of leptomeningeal enhancement on MRI of brain and/or spine performed at baseline. All patients had a histologic diagnosis of breast cancer, confirmed by review of official pathology reports. All MRIs, clinical assessments and CSF examinations were performed at our institution, and radiographic evidence of LM was confirmed by review of actual MRIs and reports.

Prior to treatment, all patients had baseline CSF cytologic examination and enhanced MRI neuroimaging of brain and spine. All patients were treated using a similar treatment regimen and schedule of assessments, modified from a prior reported study^4^ (Table 1).

**TABLE 1:** Treatment schema

Induction*	Maintenance*				
0.4 mg twice weekly X 4‐6 weeks	0.4 mg weekly X 4, then	0.4 mg every 2 wks. X 2, then	0.4 mg every mos. X 3, then	0.4 mg every 2 mos. X 3, then	0.4 mg every 3 mos.

* Dose initiated at 0.4 mg, dose reduced to 0.3 mg for grade 3+ AE after resolution

Patients received TOPO 0.4 mg twice weekly for 4‐6 weeks (induction period), then were assessed for clinical benefit by clinical examination, CSF cytology, and repeat MRI of all brain and/or spine sites which showed LM enhancement at baseline. At end of induction, patients were considered to be deriving “clinical benefit” and continued on intra‐CSF TOPO if they did not have clinical neurologic decline; unacceptable adverse events; new or progressive meningeal enhancement on MRI; or had converted from previously negative CSF cytology to positive. A small number of patients who were otherwise deriving clinical benefit but with positive CSF cytology were treated beyond induction, until either worsening of symptoms, radiographic progression or persistent positive cytology for more than 3‐4 more weeks. After induction, maintenance TOPO was administered as detailed in Table 1; CSF cytology was performed with each treatment, and clinical and MRI assessments repeated every 3 months. Treatment was continued until one or more of the following occurred: clinical neurologic progression; new or progressive leptomeningeal enhancement on MRI; conversion from negative to positive CSF cytology while on treatment; development of symptomatic progressive systemic disease warranting discontinuation; patient withdrawal; or development of unacceptable adverse events.

TOPO treatment was administered to all patients via an intraventricular reservoir. If patients required removal of the reservoir for clinical reasons, treatment was continued by lumbar puncture, until a new reservoir was placed. All patients requiring ventriculo‐peritoneal (VP) shunts had programmable valves placed in the system. The procedure for treatment in shunted patients was as follows: immediately prior to dosing, the valve was programmed to the highest position [Codman‐Hakim valves, 200; [(Codman and Shurtleff, Inc, Raynham, MA); STRATA valves, 2.5 (Medtronic, Minneapolis, MN)]. Treatment was then administered, with the valve setting at the high position for 1‐2 hours, after which the valve was restored to the pretreatment setting. The decision to utilize 1‐2 hours was based on the CSF T ½ of 1.3 hours for TOPO and active lactone metabolite,^5^ and previously reported data indicating that intraventricular drug reaches the lumbar thecal sac within 30 minutes.^9^ All patients tolerated the 1‐2‐hour period of valve adjustment. Limited field RT was provided to some patients prior to treatment. TOPO was not administered during RT, and if RT was necessary, treatment was delayed at least 2 weeks following completion of RT.

Clinical records detailing symptoms and neurologic exam findings, CSF cytopathology reports, and MRI imaging studies and reports were reviewed on all patients. Neurologic response was retrospectively defined, based on the best status achieved (improved, stable or worse) as compared with the baseline assessment. “Improved” was defined as improvement of neurologic symptoms, combined with objective improvement or stability on neurologic examination, and in the absence of CSF cytologic and/or radiographic progression. “Stable” was defined as stable neurologic symptoms and signs, in the absence of CSF cytologic or radiographic progression. Worsening was defined as any other status.

MRI response was categorized by the best status achieved (improved, stable or worse), as compared with the baseline scans, as described on the official neuroradiology reports. A designation of “improved” required that the report specifically mentioned reduction in leptomeningeal enhancement as compared to baseline, and in the absence of new areas of enhancement. “Stable” was defined as no progression or new areas of leptomeningeal enhancement. “Worse” was defined as any other status.

CSF was considered positive if the official cytopathology report described suspicious or malignant cells, and negative if containing no malignant cells, or only atypical or degenerated cells. CSF cytology was considered evaluable for response if CSF malignant cells were present at baseline, prior to TOPO treatment. Time to cytologic response (clearing) was defined as time from the first TOPO treatment to the time of the last of three consecutively negative CSF cytologic determinations. The duration of cytologic response was defined as the time from the third consecutively negative CSF cytology to the time of recurrent positive CSF cytology, or to time of cessation of TOPO for any reason.

Neurologic progression free survival (NPFS) was assessed by retrospective review of serial clinical assessments as documented, in combination with the MRI findings. Neurologic progression was defined as any of the following: worsening of neurologic examination and/or LM‐related neurologic symptoms; patient or physician decision to discontinue treatment; appearance of positive CSF cytology after durable prior negative cytology; persistently positive cytology for three consecutive determinations (with the exception of patients deriving clinical benefit without radiographic progression at end of induction); or progressive enhancement on MRI. Neurologic progression‐free survival (NPFS) was defined as the time from first intra‐CSF TOPO dose to development of neurologic progression, death due to any cause or loss of follow‐up. Overall survival (OS) was defined as the time from first intra‐CSF TOPO dose to death from any cause or loss of follow‐up.

All time‐to‐event endpoints were analyzed using the Kaplan‐Meier method, survival distributions were compared using the logrank test, and hazard ratios (HR) were calculated using Cox Proportional Hazards models. Additional exploratory analyses were performed to compare the outcomes of patients based on HER2 status (Negative vs Positive) and presence of programmable VP shunt (Not Present vs Present).

All patients signed consent for placement of intraventricular devices, and informed written consent for the intra‐CSF TOPO treatment. This case series review was approved by the Institutional Review Board.

## 3 RESULTS

A total of 31 women with breast cancer and LM received treatment with intra‐CSF TOPO. Patient demographics, ER/PR/Her‐2 marker status, and pre‐TOPO treatments were summarized (Table 2). The median time from initial diagnosis of breast cancer to LM was 84 months (range, 6‐432). The median time from LM diagnosis to first TOPO treatment was <1 month (range, <1 to 10 months); with most (90%, 28 patients) initiating treatment within 2 months of diagnosis. All patients (31/31) initially received treatment via an intraventricular reservoir. Six (19%) later received treatment via intralumbar (intrathecal) approach, following dysfunction of their intraventricular devices. Ventriculo‐peritoneal shunts with programmable valves for symptomatic increased intracranial pressure were placed in 8/31 (26%) patients. Thirteen patients (42%) had received prior RT to brain or spine ≥3 months from start of TOPO. Baseline CSF was positive for malignant cells in 22/31 (71%) and leptomeningeal enhancement on MRI was present in brain and/or spine in 27/31 (87%). Two of 10 patients with a prior history of parenchymal brain metastases had undergone surgical resection, at 2 and 4 months, respectively, prior to start of TOPO.

**TABLE 2:** Patient characteristics (N = 31)

Median Age (y, range)	58 (37‐81)
KPS (median, range)	60 (40‐100)
Median time (mos., range.), initial breast CA to LM diagnosis	84 (6‐432)
Median time (mos., range,) LM diagnosis to first TOPO	<1 (<1‐10)
Systemic cancer extent	
Regional metastases	1 (3%)
Widespread metastases	29 (94%)
No active systemic disease	1 (3%)
Prior history of brain metastases	10 (32%)
Prior surgical resection of brain metastases	2 (6%)
Marker status	
ER/PR + Her2‐	17 (55%)
Triple negative	9 (29%)
ER/PR + Her2+	3 (10%)
ER/PR‐Her2+	1 (3%)
Unknown	1 (3%)
Prior intra‐CSF methotrexate	4 (13%)
History of RT to brain or spine*	13 (42%)
WBRT	7 (23%)
SRS	1 (3%)
Spine	5 (16%)
No prior RT	18 (58%)
Positive CSF Cytology, at baseline	22 (71%)
LM enhancement on MRI, at baseline	27 (87%)
Brain only	5 (16%)
Spine only	4 (13%)
Brain and Spine	18 (55%)
None**	4 (10%)

* RT administered >3 months prior to start of TOPO.

** All pts without meningeal enhancement on brain and spine MRI had + CSF cytology for malignant cells.

The characteristics of TOPO administration, and concomitant systemic therapy and radiotherapy received are detailed in Table 3. A total of 606 TOPO doses were administered, (median per patient of 14.5, range, 3‐71). The overall median duration of TOPO treatment was 11 weeks (range, 2‐176 weeks). Twenty‐five patients (81%) remained stable and completed the induction phase, and in those patients, the median duration of TOPO treatment was 24 weeks (range, 4‐176). At data lock, 12 patients (39%) continued on therapy for ≥6 months, 8 (26%) for ≥1 year, and 4 (13%) for ≥2 years. Systemic therapy for active systemic disease was provided in 78% of patients, per treating oncologist's choice. RT was administered to 78% of patients at baseline (within 3 months of start of TOPO), to intraparenchymal brain metastases, or symptomatic areas of leptomeningeal enhancement, TOPO treatment was not co‐administered during or for 2 weeks after completion of RT. No patients received craniospinal RT.

**TABLE 3:** Treatment characteristics (N = 31)

Median # TOPO treatments (range)	14.5 (3‐54)
Patients completing induction	25/31 (81%)
Median duration TOPO (wks., range)	
All pts.	11 (2‐176)
Patients completing induction	24 (4‐176)
Concomitant systemic therapy	
Chemotherapy	14 (45%)
Hormonal therapy	9 (29%)
Her‐2 inhibitors	2 (6%)
Anti‐PD‐1	2 (6%)
Bevacizumab	2 (6%)
None	7 (23%)
Concomitant RT*	
Any	23 (74%)
WBRT	11 (26%)
SRS	8 (16%)
Focal spine	13 (42%)
None	8 (26%)

* RT administered at baseline to intraparenchymal metastases, or symptomatic areas of leptomeningeal enhancement, within 3 months of start of TOPO. TOPO treatment was not co‐administered during or for 2 weeks following completion of RT.

Response is summarized in Table 4. Best clinical response achieved was improved (13%), stable (55%), or worse (32%). The best MRI response achieved was improved (19%); stable (55%), worse (16%), or not known (10%—follow‐up MRI not done). All six patients with improvement on MRI had received limited field RT. In comparison of MRIs with radiation treatment fields, four patients had received RT to the sites of improvement, and two patients also had objective decrease in enhancement in areas outside the treatment fields.

**TABLE 4:** Response (N = 31)

# Pts. clearing CSF malignant cells	10/21 (48%)
Med duration, CSF clearing (months, range)	10.2 (1.4‐34.5)
Med time to CSF clearing (months, range)	1.1 (0.2‐2.3)
Best clinical response	
Improved	4 (13%)
Stable	17 (55%)
Worse	10 (32%)
Best MRI response	
Improved*	6 (19%)*
Stable	17 (55%)
Worse	5 (16%)
Not done	3 (10%)
Median NPFS, months (range)	
All pts.	2.5 (0.2‐40.5)
Pts. Compl. Induction**	5.5 (0.9‐40.5)
NPFS6, % (95% CI)	
All pts.	38.7% (24.9%‐60.3%)
Pts. Compl. Induction**	48.0% (31.9%‐72.2%)
Median OS months ( range)	
All pts	6.9 (0.9‐48.8)
Pts. Compl. Induction**	11.5 (1.8‐48.8)
OS6, % (95% CI)	
All pts	54.8% (39.8%‐75.5%)
Pts. Compl. Induction**	68.0% (52.0%‐89.0%)
OS12, % (95% CI)	
All pts.	35.5% (22.1%‐57.0%)
Pts Compl. Induction**	44.0% (28.3%‐68.5%)
OS24, % (95% CI)	
All pts.	19.4% (9.4%‐39.7%)
Pts. Compl. Induction**	24.0% (11.9%‐48.2%)

NPFS, neurologic progression‐free survival; OS, overall survival.

* 2/6 pts. had decrease in leptomeningeal enhancement outside of RT treatment fields.

** Pts. Compl. induction = patients completing 4‐6 weeks (8‐12 doses) of induction TOPO.

Of 21 (68%) evaluable patients with positive CSF cytology at baseline, 10 (48%) cleared CSF of malignant cells for at least three consecutively negative CSF specimens, meeting our definition for CSF response. The median time from start of TOPO to conversion of CSF cytology from positive to negative was 4 weeks (range, 1‐10 weeks). In the patients clearing CSF of malignant cells, the median duration of CSF cytologic clearing was 15.9 months (range, 1.4‐34.5 months). Two patients initially cleared CSF of malignant cells for 69 and 48 weeks, respectively, but later relapsed; one during treatment interruption for replacement of a ventricular device, and one after entering the every 3‐month maintenance period. These patients were re‐challenged with intraventricular TOPO with induction dosing, and both cleared CSF of malignant cells again, at 6 and 1 weeks, respectively, with CSF remaining negative for malignant cells on TOPO for 105 and 49 additional weeks, respectively.

CSF CA 15‐3 levels were measured in three patients at baseline; all had positive CSF cytology also at baseline. In all three, CSF cleared of malignant cells during TOPO treatment and serial CA15‐3 levels decreased below the limits of detection of the assay (<8 U/ml).

**FIGURE 1:**
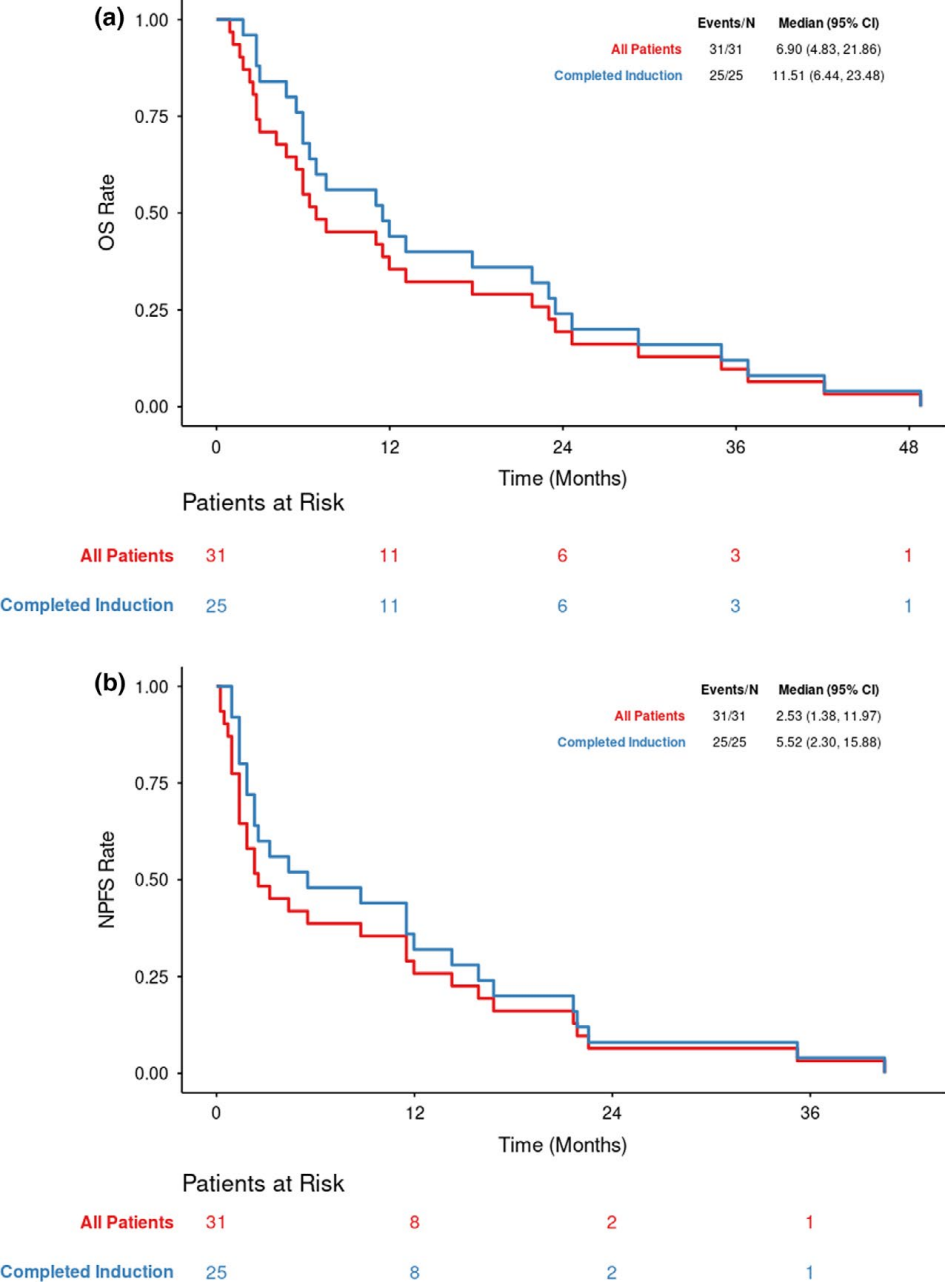
A, Overall survival. B, Neurologic progression‐free survival

Survival outcome is presented in Figure 1. Median OS for all patients was 6.9 months (range, 0.9‐48.8 months), and for the subgroup completing induction, 11.5 months (range, 1.8‐48.8 months). OS was shorter in patients with HER‐2‐positive tumors, compared with those with HER‐2‐negative disease (HR = 5.44, 95% Confidence Interval (CI)=1.63‐18.14, logrank‐*P* = .002), as well as in patients with programmable VP shunts, compared to those without shunts (HR = 2.1, 95% CI = 0.89‐4.98, logrank‐*P* = .084), though this latter comparison was underpowered due to small sample size. OS6, OS12, and OS24 are detailed in Table 4. Median neurologic PFS (nPFS) for all patients (N = 31) was 2.5 months (range, 0.2‐40.5 months) and for those completing induction therapy (N = 25), 5.5 months (range, 0.9‐40.5 months). Neurologic progression, based on worsening clinical symptoms or signs, CSF cytologic relapse, or worsening on MRI was eventually documented in 27/31 (87%) patients. The remaining four patients (13%) did not meet criteria for neurologic progression, but were taken off intra‐CSF therapy either for systemic progression (2), or for death from systemic disease without documented neurologic progression (2). Similar to the OS results, nPFS was also shorter in patients with HER‐2‐positive tumors (HR = 5.45, 95% CI = 1.79‐16.56, logrank‐*P* < .001) and in patients with programmable VP shunts (HR = 1.7, 95% CI = 0.75‐3.89, logrank‐*P* = .20), though this latter comparison was underpowered due to small sample size.

Adverse events included reversible headache (3), vomiting (2) in the first 2 days following treatment. For subsequent cycles these patients were premedicated with antiemetics and dexamethasone, TOPO dose reduced to 300 micrograms, without headache or vomiting.. Two (6%) patients developed bacterial meningitis requiring removal of the intraventricular reservoir; after recovery, treatment was resumed by intralumbar approach. One patient developed incisional dehiscence at the site of the intraventricular device. Two patients developed increasing T2 hyperintensity around the intraventricular catheter, and treatment was subsequently administered intralumbar. Two additional patients discontinued treatment due to unrelated adverse events (1‐systemic infection; 1‐ pulmonary embolus). We did not identify hematologic toxicity attributable to intra‐CSF TOPO.

## 4 DISCUSSION

Topotecan has shown activity in treatment of breast cancer.^2^, ^3^ In preclinical and clinical studies, TOPO has demonstrable radiation‐sensitizing effects in multiple solid tumor histologies, including breast carcinoma,^10^, ^11^ and in patients with brain metastases.^10^, ^12^, ^13^ Although the mechanism is unclear, the radiosensitizing effect may relate to accumulation of cells in S phase and inhibition of DNA repair.^10^ When topotecan is administered by intraventricular route in humans at the MTD of 0.4 mg, the AUC is 21 micromolar‐hour, T 1/2α is 26 ± 9 minutes, and T 1/2β is 174 ± 72 minutes.^5^, ^10^, ^14^

Following intra‐CSF treatment of patients with LM and breast cancer, survival has typically ranged from 2.9‐5.4 months,^4^, ^15^, ^16^, ^17^, ^18^, ^19^, ^20^, ^21^, ^22^, ^23^, ^24^ and up to 13.6 months in HER2+ patients treated with intra‐CSF trastuzumab.^25^ Prior studies with intra‐CSF TOPO treatment have reported clearing of malignant CSF cells in 38% of children with refractory leptomeningeal leukemia^6^ and 82% (9/11) of patients with primary CNS tumors^8^; however, the duration of CSF clearing in those studies was not reported. In a subgroup of 19 patients with breast cancer and LM, intra‐CSF TOPO was associated with a median TTP of 6 weeks (95% CI: 5, NR) and median OS of 13 weeks (95% CI: 11,32).^4^

In our study, most patients (94%) had advanced stage disease and had received multiple prior systemic treatment regimens at time of LM diagnosis. Despite this, nearly one‐half of patients with initially positive CSF cytology cleared CSF of malignant cells, which was reasonably durable (median, 10 months; 60% >6 months, 40% >12 months). CSF clearing was observed relatively early on, at a median of 4 weeks. The observed 45% CSF cytologic response rate compares favorably with that previously reported (21%) with TOPO treatment of patients with LM and unselected malignancies.^4^ Not surprisingly, patients who remained progression‐free through the induction period (4‐6 weeks) appeared to derive the most benefit. The median OS and median neurologic PFS for these patients were 11.5 months and 5.5 months, respectively, compared with 6.9 months and 2.5 months when including those not completing induction. Of those completing induction, over two‐thirds were alive at 6 months, and almost half of such patients (44%) remained stable on TOPO for nearly a year. Based on this observation, it is proposed that patients who remain at least stable and complete induction are more likely to derive benefit from treatment beyond induction. Activity of TOPO was also suggested by the observations of second cytologic response with TOPO re‐challenge in patients experiencing CSF cytologic relapse, and by the reduction in initially elevated CSF CA 15‐3 to undetectable levels in three patients, all of whom also showed clearing of CSF malignant cells.

The majority (52%) of our patients with LM had ER+ PR+, Her‐2‐negative neoplasms, 16% were Her‐2 positive, and 26% were triple‐negative, which is similar to the proportions reported by others.^25^, ^26^, ^27^, ^28^, ^29^, ^30^, ^31^ Although numbers were small, we observed that OS was shorter in Her‐2 positive as compared with Her‐2‐negative patients (HR 5.44, *P* = .0059). Of possible relevance was that only two of the four Her‐2+ patients were receiving systemic Her‐2 inhibitors.

The authors recognize the inherent limitations of a retrospective case series review with a modest sample size. It is conceivable that our study population included a disproportionate number of patients harboring tumors with indolent biological behavior; notable was that the median time from initial breast cancer diagnosis to LM diagnosis was 7.0 years in our population. Since this parameter has not typically been reported in prior series, it is difficult to make comparisons. It is likely that local‐field radiation therapy at baseline for bulky or symptomatic disease (74%), concomitant hormonal (50%) or systemic chemotherapy (46%) might have influenced outcome; however, it is noted that these concomitant therapies have been provided in conjunction with intra‐CSF therapy in previously reported studies.^4^, ^17^, ^18^, ^19^, ^21^, ^22^, ^27^, ^28^, ^29^, ^30^, ^31^, ^32^ Given that 74% of our patients received local field RT at baseline, we acknowledge that the results likely reflect that achievable with the combination of RT (when clinically warranted) followed by intraventricular topotecan, and not just due to topotecan alone. In our study, the observed neurologic PFS6 of 39% and median OS 6.9 months are notable, particularly when compared with prior reports involving breast cancer patients with LM treated with a variety of systemic or intra‐CSF agents, given alone or in combination with systemic therapy and/or radiotherapy. In those reports, PFS6 was reported of 26%, and median OS, 3.3‐5.4 mo).^17^, ^18^, ^19^, ^20^, ^21^, ^22^, ^23^, ^24^, ^25^, ^27^, ^28^, ^29^, ^30^, ^31^, ^32^ The number of long‐term survivors in our group (OS12, 35.5%, OS24, 19.4%) also compares favorably with that previously reported(OS12, 8%‐31%).^19^, ^22^, ^23^, ^31^ Note is made that preliminary data for intra‐CSF trastuzumab in treatment of Her2 + LM patients has been promising (median PFS, 5.7‐7.5 months; PFS6 = 41%; median OS, 13.5‐10.6 months).^20^, ^21^

## 5 CONCLUSION

Intra‐CSF TOPO, alone or in combination with focal RT has activity in breast cancer patients with LM, producing durable clearing of CSF malignant cells in nearly one‐half of patients, notable neurologic PFS6 and median OS, and a number of long‐term survivors. Intra‐CSF TOPO was generally well tolerated. The results support further testing within the context of a prospective trial.

## CONFLICT OF INTEREST

The authors declare no conflicts of interest regarding work involved in this study or manuscript.

## AUTHOR CONTRIBUTIONS

Kurt A. Jaeckle involved in conceptualization, methodology, validation, investigation, data curation, writing, review, and editing of the manuscript. Jesse Dixon and Stephen Keith Anderson involved in methodology, data analysis, writing, review, and editing of the manuscript. Kathy Hebenstreit carried out investigation, data curation, and review of the manuscript. Alvaro Moreno‐Aspitia carried out investigation, review, and editing of the manuscript. Geraldo Colon‐Otero, Samarth L. Reddy, Tejal Patel, and Edith Perez contributed to investigation and review of the manuscript.

## Data Availability

Data available on reasonable request from the author.
